# Supplementary material for: Deletion of Diterpenoid Biosynthetic Genes CYP76M7 and CYP76M8 Induces Cell Death and Enhances Bacterial Blight Resistance in Indica Rice ‘9311’
Source: Int J Mol Sci. 2022 Jun 29;23(13):7234. doi: 10.3390/ijms23137234 (PMC9266670; doi:10.3390/ijms23137234)
Supplement: Supplementary file 1 [file ijms-23-07234-s001.zip › ijms-1763551-supplementary.pdf]

Supplementary materials

Supplementary Figure S1.. ROS accumulation in *g380* before the lesions formation. (a) DAB staining of *9311* and *g380* leaves. (b-f) The ROS related physiological-biochemical indexes of *9311* and *g380* leaves. Bar=1 cm in (a). Values are mean ± SD (n = 3). ** indicates significant differences at p<0.01 level by Welch's t-test, * indicates significant differences at p<0.05 level by Welch's t-test, *ns* indicates p>0.05 level by Welch's t-test.

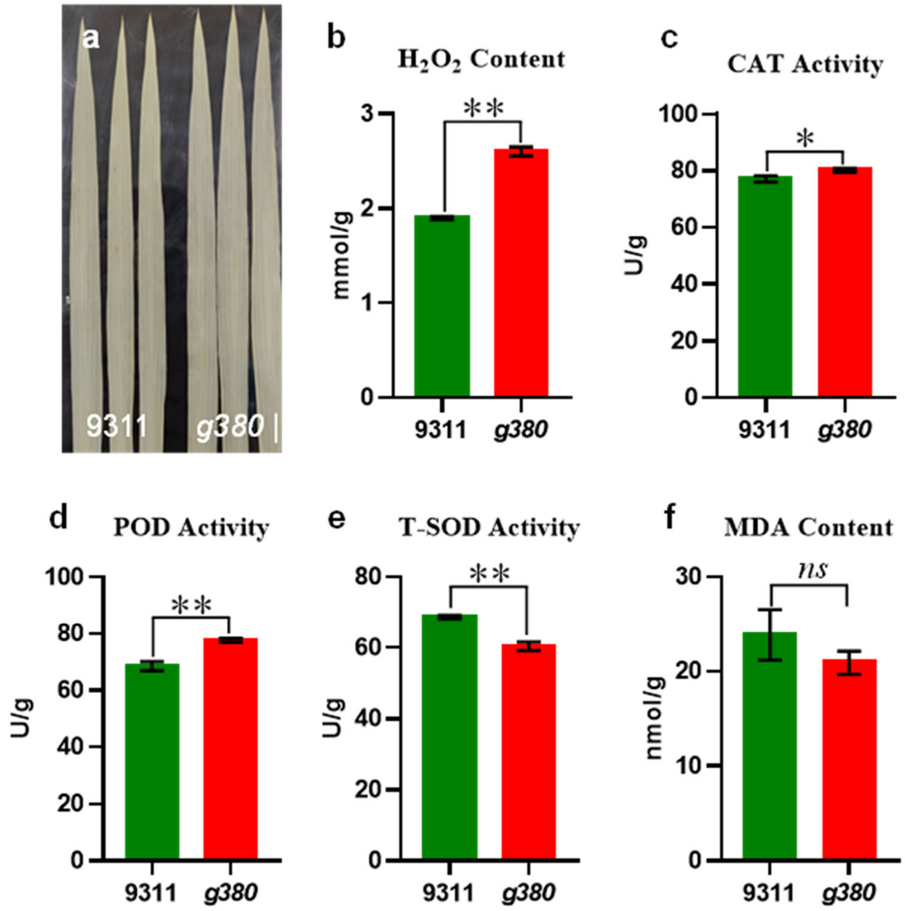

Supplementary Figure S2. Upregulated genes enriched the phenylpropanoid biosynthesis (dosa00940) pathway.

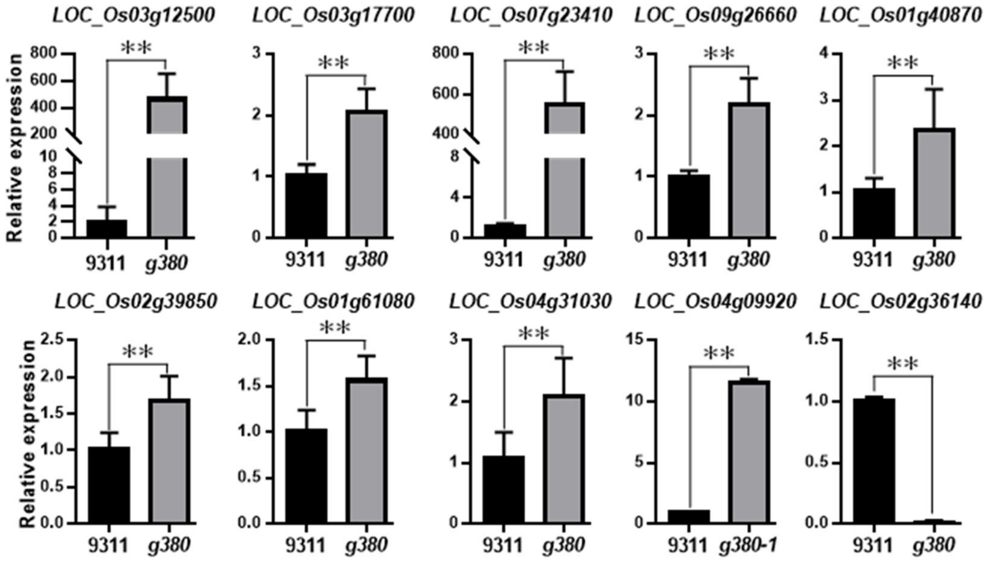

Supplementary Figure S3. Lignin content of *9311* and *g380* before *g380* lesion formed.

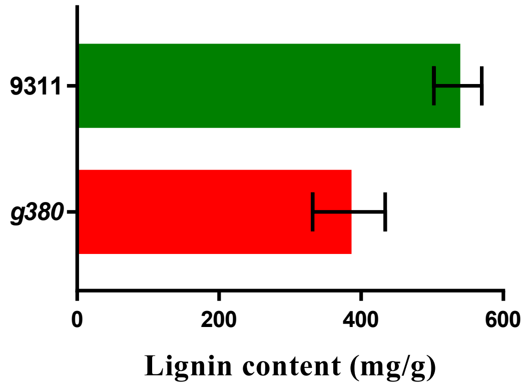

Supplementary Table S1. Summary of RNA-seq data production.

Sample	Raw reads	Raw bases	Clean reads	Clean bases	Clean data rate	Reads mapped to genome	Mapping rate
*g380*_1	109,394,138	10,939,413,800	102,335,692	10,012,335,791	91.53%	79,424,324	77.61%
*g380*_2	99,866,956	9,986,695,600	93,789,966	9,195,128,262	92.07%	72,140,490	76.92%
*g380*_3	103,030,918	10,303,091,800	97,158,506	9,535,482,959	92.55%	73,454,724	75.60%
9311_1	112,525,000	11,252,500,000	103,597,194	10,141,888,103	90.13%	81,316,532	78.49%
9311_2	102,858,738	10,285,873,800	97,198,894	9,547,028,366	92.82%	70,210,316	72.23%
9311_3	116,315,728	11,631,572,800	109,122,984	10,710,401,300	92.08%	83,746,156	76.74%

Supplementary Table S2. Differentially expressed *PR* genes.

gene_symbol	**MSU_ID**	**log_2FC**	***P* Value**	**FDR**
OsPR1b	LOC_Os01g28450	6.09	2.76E-09	9.66E-07
OsPR1a	LOC_Os07g03710	8.18	3.72E-12	2.45E-09
PR5	LOC_Os12g43430	5.14	5.03E-14	4.34E-11
PR1c	LOC_Os01g28500	2.89	3.91E-06	7.30E-04
PR1	LOC_Os07g03730	5.43	8.42E-09	2.72E-06
Gns5	LOC_Os01g71340	5.07	7.58E-09	2.51E-06
Gns6	LOC_Os01g71350	4.28	8.73E-14	7.41E-11
OsCHIT8	LOC_Os06g51060	3.52	3.88E-05	5.36E-03
OsCHIT14	LOC_Os10g39680	3.54	5.00E-05	6.62E-03
OsCHIT17	LOC_Os05g33130	6.94	9.61E-14	8.03E-11
OsPR4a	LOC_Os11g37970	3.94	1.62E-09	5.94E-07
OsPR4c	LOC_Os11g37950	7.41	2.74E-14	2.48E-11
TLP	LOC_Os03g46070	4.27	4.86E-17	6.80E-14
BBTI-7	LOC_Os01g03390	5.42	1.40E-09	5.21E-07
BBTI-12	LOC_Os01g04050	2.56	3.51E-05	4.92E-03
Oschib1	LOC_Os10g28080	5.09	5.20E-18	9.40E-15
OsPR10	LOC_Os12g36880	5.54	1.01E-15	1.16E-12
PR10B	LOC_Os12g36850	5.51	2.47E-15	2.61E-12
RSOsPR10	LOC_Os12g36830	5.44	2.07E-10	9.20E-08
OsGLP8-7	LOC_Os08g09010	3.39	4.50E-04	4.34E-02

Supplementary Table S3. Differentially expressed *WRKY* genes.

gene_symbol	MSU_ID	log_2FC	*P* Value	FDR
OsWRKY14	LOC_Os01g53040	3.04	1.38E-04	1.64E-02
OsWRKY24	LOC_Os01g61080	2.44	1.20E-04	1.44E-02
OsWRKY32	LOC_Os02g53100	4.79	3.06E-05	4.42E-03
OsWRKY19	LOC_Os05g49620	4.59	8.63E-16	1.03E-12
OsWRKY28	LOC_Os06g44010	3.71	7.77E-11	3.99E-08
OsWRKY69	LOC_Os08g29660	2.70	3.96E-05	5.44E-03
OsWRKY72	LOC_Os11g29870	3.21	1.15E-07	2.97E-05

Supplementary Table S4. Upregulated genes enriched the phenylpropanoid biosynthesis (dosa00940) pathway.

gene_symbol	**MSU_ID**	**log_2FC**	***P* Value**	**FDR**
prx15	LOC_Os01g22352	4.47	6.40E-12	3.89E-09
prx16	LOC_Os01g22370	1.65	2.60E-04	2.74E-02
OsALDH2C1	LOC_Os01g40870	2.37	3.77E-11	2.03E-08
prx22	LOC_Os01g73200	2.09	4.88E-05	6.49E-03
Os4CL3	LOC_Os02g08100	1.81	3.38E-05	4.79E-03
OsCAD2	LOC_Os02g09490	3.21	2.07E-08	6.36E-06
OsHCT2	LOC_Os02g39850	2.12	6.60E-09	2.20E-06
OsPAL1	LOC_Os02g41630	3.44	3.40E-09	1.18E-06
OsPAL2	LOC_Os02g41650	2.43	8.77E-06	1.52E-03
OsPAL4	LOC_Os02g41680	3.47	5.76E-12	3.58E-09
OsBGLU1	LOC_Os03g53800	2.70	1.38E-06	2.91E-04
OsHCT1	LOC_Os04g42250	1.77	1.34E-06	2.85E-04
prx61	LOC_Os04g59190	4.63	9.48E-07	2.07E-04
prx117	LOC_Os08g02110	4.55	7.56E-17	1.01E-13
OsCOMT	LOC_Os08g06100	2.58	9.99E-08	2.68E-05
OsCCR20	LOC_Os08g34280	2.65	2.31E-07	5.61E-05
Os9BGlu31	LOC_Os09g33680	1.05	3.37E-04	3.42E-02
OsF5H1	LOC_Os10g36848	4.20	2.04E-17	3.31E-14

Supplementary Table S5. Upregulated genes enriched the plant-pathogen interaction (dosa04626) pathway.

gene_symbol	**MSU_ID**	**log_2FC**	***P* Value**	**FDR**
OsPR1b	LOC_Os01g28450	6.09	2.76E-09	9.66E-07
PRB1-2	LOC_Os01g28500	2.89	3.91E-06	7.30E-04
OsWRKY24	LOC_Os01g61080	2.44	1.20E-04	1.44E-02
KCS	LOC_Os02g11070	2.59	3.63E-09	1.25E-06
OsCEBiP	LOC_Os03g04110	1.32	3.49E-04	3.52E-02
OsMPK3	LOC_Os03g17700	2.51	4.70E-07	1.09E-04
OsRIN4	LOC_Os04g31030	1.56	2.16E-04	2.36E-02
OsCML21	LOC_Os05g24780	3.81	5.52E-08	1.59E-05
OsPR1a	LOC_Os07g03710	8.18	3.72E-12	2.45E-09
Osrboh7	LOC_Os09g26660	2.71	2.10E-04	2.32E-02
OsPR1-101	LOC_Os10g11500	4.34	3.05E-04	3.15E-02
OsCML36	LOC_Os11g04560	3.10	4.78E-07	1.10E-04

Supplementary Table S6. Sequences of primers for linkage analysis and chromosome walking.

Primer ID	Forward (5′-3′)	Reverse (5′-3′)
RM341	CAAGAAACCTCAATCCGAGC	CTCCTCCCGATCCCAATC
C2-9	TTTTTATCTCGAGGTACGGTAGTAG	TGAGAATTAGTGTTCATTTGTAGATTC
C2-10	GGCTGGCTGTTGCTCATC	AAAAATCCCAACCCTGCTG
RM13611	CTCTTGAACGGCTGCACGAAAGG	CGCGAGAATGGTAGGTGGATCG
RM3850	AAGTTGAGAATGAGGGACAA	TTCGGAAGTGAAAAGGTAAT
L24	CCTCCTCCTGAAAATCCA	GTACCACCTCGTCGTCGC
L26	GAGGTTTGTGATTGGTCG	ATCGTTGCTTGAAGTTGA
L39	CTATTCCTATTCCACCAGC	TCGTGCGATAACACTAAA
L45	GCAAGTTTTAAGTACGATC	TACAGTTGTGAACACCCA
L46	AGCGACCGAATTGAAGAG	TAAGCCGTGGCAAGGAAC
L4	GCGATTTGACCGTCCTTT	GACTTCCCGACACCTGCT
L6	GCTTACTACCGTACCACAT	AGGCTCCATTCTTCCATA
L7	ACTGTCACCCTGATTTGC	GCTGCCTAAGTGATTATTTC
L9	CTCGCTAAGTATGAGATTTG	GGTTCTTGATTACGGAGG
L10	TGGCTGTTTGCTTGTATC	GAAATGCTGTAAGCGAGA
L12	GAGCGTAGGAGCAAACAG	TGAGATAGAACTCAGGGAG
L14	CAGTGACCGTTGTTTGTT	GGATTATTAGGTTGGCTTTT
L15	CCCTAGACACGTACAAAA	CAAACTTATTCAAGCAGAT
L16	GCCACTGCCCAAATCCTC	TTGCCCCTCGTTTTACCA
L27	TAAGTAATTGTGGCTTCCTT	CCGTAGTTCTATAATATGGG
L28	TTTTCAAACTAATGGTCT	TAAGAAGGTAGTGACAGC
L34	TGTGCTTGCTGTGCCTCT	GCCGATTTTGGGATTTGT
L36	TCTGGCTATGGTTTGCT	TATTTGTCACCGTGGGT
L37	TTTTCATAGTCGTTGGC	GCACTAGCATCTAGCGT
L38	ACGCTACTTTTCTTGCT	GACCGTCCGTCTTATTT
L40	ATTGCCAGGCTATGTTTC	TGCGATATTGCATCTCAT
F11	TTCTAACCAGCACCACACAAT	GTAGCCATGACACAACCATCT
F12	GCATACTGAGATGGTTGTG	TGGTGGGTAATGAATAGAG
F13	GTCTGGATGATGCAAGGGA	CCAACACGATAATGGGGAA
F4	GAAGATTGATTGTGTTTGA	CTAGATGCTGTATGGTTGA
F5	ACCAATCAACCATACAGCA	GAGCATCATCAGCATCACC
R8	ATGAGGTGAAAAAAGGAAAATG	TCTATAGGGTCCCAACCAAGGT
R9	TTGCGTCCTTCATTTTCCTATT	GCCATTTTCCTTTTTTCACCTC
R10	AGAATACAAAAGGTAGCAAC	TCAATAGGAAAATGAAGGAC
R11	AGAAATACGGAGCCGACGGAAAT	AGGGGAAGAAGGAAGGTAGGGAG
R12	GCGGTTCACTAATGATAGAAAAG	CAATGGTAATAGAAACACGGATA

Supplementary Table S7. Sequences of qRT-PCR primers.

Gene symbol/ID	Forward (5′-3′)	Reverse (5′-3′)
Actin	CAGGCCGTCCTCTCTCTGTA	AAGGATAGCATGGGGGAGAG
OsPR1a	GGCCAATCTCCCTACTGATTAA	GCATAAACACGTAGCATAGCAT
OsPR1b	TACGCCAGCCAGAGGAGC	GCCGAACCCCAGAAGAGG
OsPR10	GGTGTGGGAAGCACATACAA	GTCTCCGTCGAGTGTGACTTG
OsCPS2	CATCTCCAAGGTTTTGTTCGAG	TTTTCGCAGTCATCACTTTACG
CYP76M6	GGAAAGACTACGAGTACATCCC	AACTTCTCAGTCATGTCCATGT
OsCPS4	ATCTACCCTTTGGATGTGTACG	TGTCATCGATATCCTTTACCGG
OsKSL4	TTTGAGGTCTTTAGCGACAGAT	TATAGTTGGTCCCAATGCGAAT
CYP99A2	ATCATGGAGATAAACTCGGCAG	CTCTTCTTTGACGACGACTTG
OsPAL1	GACCCTGTATTTTCTTCGTTCG	AGTAGCAATACTTTCACCCCAA
OsPAL4	GTGCTCTTTGAGGCTAACATTC	CTTCTTGGCATGCTTCATGTAG
OsF5H1	TTCAACATCGGCGACTTCATC	ATGATCTTGTCGATGAACCTGT
OsROMT9	GAAGAACCACTCCGTCATCATC	GAGGATCCACTTCATCAGGATG
OsCCR20	ATGGCGTACGTTAATTAGTTGC	TTTCCTTGCAGTAGTCAAGGTC
OsCAD2	GTACTGCAACAAGAGGATTTGG	GATCTTCACCACAAACTTCTGG
LOC_Os03g12500	AAGCTGCTGCAATACGTGTACTGG	CGACGAGCAACAGCCTTCCG
LOC_Os03g17700	TTACCTAGAGAGATTGCACGAC	TGGTGATTCAATCTAGTACCGG
LOC_Os07g23410	TACTTCTCGTGGAAATACACCC	TTGTACTTGTACACGTATGGGG
LOC_Os09g26600	GAGAGGAGCTCTACGACTTCT	CTGTACTTCCTCCCTCGTTATC
LOC_Os01g40870	GTGACCAAGAACCTCAACATTG	TGGTACATGGTACACTCTAGCT
LOC_Os02g39850	AGCCCACCAAGCTGTACT	GAAGATCACGTTGCCGAAGTAG
LOC_Os01g61080	GCCGACGACCAGCACGATCACC	ACGAGCCGACGCCGCCCTC
LOC_Os04g31030	CTACAGGATTGGAAGTCGCTAA	GCATTCTCAAACTTCTGTGTGT
LOC_Os04g09920	CTCAAGTTCAGCAATGGGTTC	CCAGTATAATTGCCTTGACGTG
LOC_Os02g36140	CGAAGAATACATGGAAAGTGGC	GCCTGAACAACTCATCGTATTC
